# A Mobile Element in *mutS* Drives Hypermutation in a Marine *Vibrio*

**DOI:** 10.1128/mBio.02045-16

**Published:** 2017-02-07

**Authors:** Nathaniel D. Chu, Sean A. Clarke, Sonia Timberlake, Martin F. Polz, Alan D. Grossman, Eric J. Alm

**Affiliations:** a Microbiology Graduate Program, Microbiology Graduate Program, Massachusetts Institute of Technology, Cambridge, Massachusetts, USA; b Center for Microbiome Informatics and Therapeutics, Massachusetts Institute of Technology, Cambridge, Massachusetts, USA; c Department of Biological Engineering, Massachusetts Institute of Technology, Cambridge, Massachusetts, USA; d Department of Civil and Environmental Engineering, Massachusetts Institute of Technology, Cambridge, Massachusetts, USA; e Department of Biology, Massachusetts Institute of Technology, Cambridge, Massachusetts, USA; University of Georgia

## Abstract

Bacteria face a trade-off between genetic fidelity, which reduces deleterious mistakes in the genome, and genetic innovation, which allows organisms to adapt. Evidence suggests that many bacteria balance this trade-off by modulating their mutation rates, but few mechanisms have been described for such modulation. Following experimental evolution and whole-genome resequencing of the marine bacterium *Vibrio splendidus* 12B01, we discovered one such mechanism, which allows this bacterium to switch to an elevated mutation rate. This switch is driven by the excision of a mobile element residing in *mutS*, which encodes a DNA mismatch repair protein. When integrated within the bacterial genome, the mobile element provides independent promoter and translation start sequences for *mutS*—different from the bacterium’s original *mutS* promoter region—which allow the bacterium to make a functional *mutS* gene product. Excision of this mobile element rejoins the *mutS* gene with host promoter and translation start sequences but leaves a 2-bp deletion in the *mutS* sequence, resulting in a frameshift and a hypermutator phenotype. We further identified hundreds of clinical and environmental bacteria across *Betaproteobacteria* and *Gammaproteobacteria* that possess putative mobile elements within the same amino acid motif in *mutS*. In a subset of these bacteria, we detected excision of the element but not a frameshift mutation; the mobile elements leave an intact *mutS* coding sequence after excision. Our findings reveal a novel mechanism by which one bacterium alters its mutation rate and hint at a possible evolutionary role for mobile elements within *mutS* in other bacteria.

## IMPORTANCE

DNA mutations are a double-edged sword. Most mutations are harmful; they can scramble precise genetic sequences honed over thousands of generations. But in rare cases, mutations also produce beneficial new traits that allow populations to adapt to changing environments. Recent evidence suggests that some bacteria balance this trade-off by altering their mutation rates to suit their environment. To date, however, we know of few mechanisms that allow bacteria to change their mutation rates. We describe one such mechanism, driven by the action of a mobile element, in the marine bacterium *Vibrio splendidus* 12B01. We also found similar mobile genetic sequences in the *mutS* genes of many different bacteria, including clinical and agricultural pathogens. These mobile elements might play an as yet unknown role in the evolution of these important bacteria.

## INTRODUCTION

To adapt to changing environments—whether changes in pH or exposure to antibiotics—bacteria rely on mutations to produce new genetic variants that can survive under new conditions. The majority of possible mutations, however, are deleterious, so all organisms face a trade-off between genetic fidelity, which enables accurate gene replication, and genetic innovation, which provides new genetic diversity (1). Observed mutation rates in bacteria studied in the laboratory are thought to balance this trade-off, because a wide range of microorganisms have similar, low mutation rates: about one mutation per genome per 300 cell divisions (2).

Nevertheless, bacterial strains with higher than expected mutation rates—known as mutators—have been found in diverse habitats. These habitats include the respiratory tracts of patients with cystic fibrosis (3, 4), the mouse gut (5), and the human gut and urinary tract (6–8). In clinical settings, mutator bacteria are believed to contribute to the rise of antibiotic resistance, which is increasingly recognized as a critical burden on global health (9, 10). In seeking to reconcile the prevalence of bacterial mutators with the deleterious nature of mutations, many past theoretical studies found that constitutive mutators—bacteria with a fixed, elevated mutation rate—can be maintained at low frequencies in asexual populations (11–13). But even though mutator cells as a group can persist in a community, each mutator cell can pass on its genes only by reverting to a nonmutator state or by recombining its genes with nonmutator members of the community via horizontal gene transfer (14). Otherwise, unchecked, rapid accumulation of mutations would make each mutator cell an evolutionary dead end, a paradigm akin to Mueller’s ratchet (15).

It has been proposed that many bacteria might resolve the fidelity versus innovation dilemma by altering their mutation rates. One strategy involves active regulation of mutation rate, or stress-induced mutagenesis (16). In this strategy, bacteria respond to stress by increasing their mutation rates (17). Such bacteria can thus harness genetic innovation during periods of environmental change—like the application of antibiotics—while maintaining genetic fidelity during periods of stability; their mutation rate is thus actively linked with a need to adapt. This active strategy is distinct from stress-driven mutagenesis, where a given stress triggers a change in mutation rate irrespective of active bacterial response (e.g., UV radiation) (17).

Despite this theoretical framework, few mechanisms are known that allow bacteria to alter their mutation rates, and the mechanisms uncovered to date have largely been constrained to well-studied model organisms. One mechanism that increases mutation rates is upregulation of genes encoding error-prone DNA polymerases, which introduce more errors than typical polymerases (18, 19). Another mechanism is downregulation of genes in the mismatch repair pathway, a set of genes that encode proteins that proofread DNA during replication, recombination, and damage (20). Most described mutator bacteria with deficits in mismatch repair genes have partial or full loss-of-function mutations in one of the mismatch repair genes (e.g., *mutS*, *mutL*, or *mutH*) (7, 21–24). Researchers have previously observed elevated rates of polymorphism and recombination in this chromosomal region, which in many bacteria also includes the stress response gene *rpoS* (25, 26). Such variation and instability have led some to propose that *mutS* is a “contingency gene,” a highly mutable locus that allows an organism to rapidly alter its genotype (27).

To our knowledge, only three cases of mismatch repair mutations have been described that do not involve constitutive or loss-of-function mutations and thus a constitutive mutator phenotype. Some strains of *Escherichia coli* downregulate *mutS* expression during the transition to stationary-phase growth, increasing their mutation rate by an order of magnitude (28). Strains of *E. coli*, *Pseudomonas aeruginosa*, and *Vibrio cholerae* also downregulate *mutS* via an *rpoS*-dependent response to antibiotics (29). Strains of *Streptococcus pyogenes* contain a prophage that, when integrated into the bacterial chromosome between *mutS* and *mutL*, halts transcription of *mutL*, increasing the mutation rate 100-fold (30). The prophage excises itself during exponential growth and reintegrates itself when cells reach the stationary phase (30). Thus, integration and excision of this prophage cause a temporary and reversible increase in mutation rate in response to the environmental stress of entering into stationary phase.

Here, we report a mechanism that allows *Vibrio splendidus* 12B01, a common marine bacterium, to increase its mutation rate. We also identified diverse mobile elements within the *mutS* sequences of hundreds of environmental and clinical bacteria, suggesting that these elements could play a role in regulating mutation rates in many bacteria.

## RESULTS

### Whole-genome sequencing revealed elevated, variable, and transition-biased mutation rates in *V. splendidus* 12B01.

During serial selection for salt-tolerant mutants in *V. splendidus* 12B01 (see Materials and Methods and Fig. S1 in the supplemental material), we discovered a mobile element that alters the bacterium’s mutation rate. We performed whole-genome sequencing on 40 isolated colonies from eight independent bacterial lineages (lineages 1 to 8), which originated from the same ancestral strain over five rounds of selection on high-salinity plates (data access, NCBI BioSample no. SAMN05560410). We identified *de novo* mutations by calling high-confidence single-nucleotide polymorphisms (SNPs) between each genome assembly and a reference genome for the ancestral strain (see Materials and Methods). We checked for contamination by confirming that each strain maintained all mutations that arose in the previous selection round. We also quantified deletions, insertions, and inversions but focused on SNPs in further analyses because they were more common, easier to quantify, and simple to compare across lineages.

10.1128/mBio.02045-16.1 Fig. S1  Hypersalinity selection experimental design. Each colony growth on a plate corresponded to ~24 generations, and each liquid medium culture corresponded to ~8 generations. Download Fig. S1, PDF file, 0.1 MB. Copyright © 2017 Chu et al. 2017 Chu et al. This content is distributed under the terms of the Creative Commons Attribution 4.0 International license.

**FIG 1:**
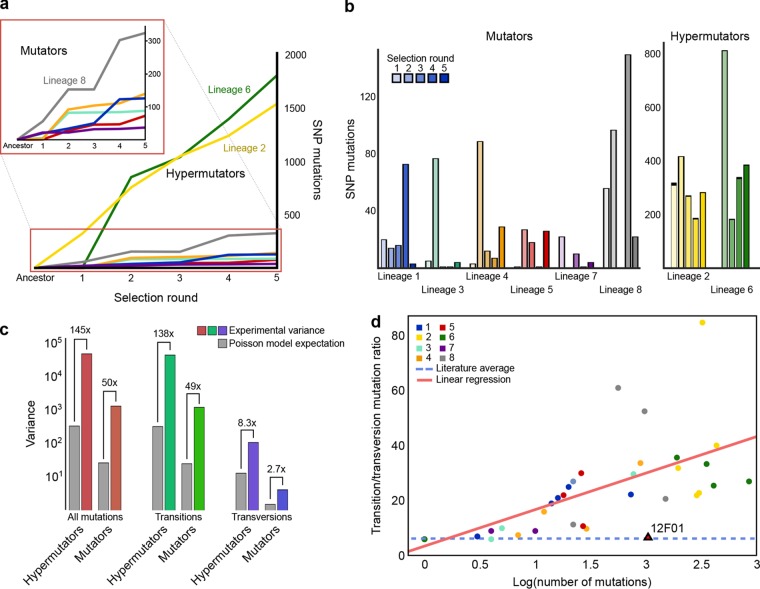
Serial selection for salt tolerance identifies a hypermutator phenotype, with a distinct mutation profile, in *Vibrio splendidus* 12B01. (a) We grew eight independent lineages on hypersaline media and sequenced genomes from each selection round. Strict SNP calling indicated that two hypermutator lineages had rapidly accumulated a large number of mutations, but all lineages had accumulated many more mutations (37 to 1,802) than the one or two mutations expected, given literature averages of spontaneous mutation (2). (b) The number of new mutations varied greatly across selection rounds in both mutator and hypermutator lineages. (c) This variability was much higher than the expected Poisson distribution variance, and this disparity was largely driven by transition mutations. (d) Selection rounds with more mutations tended to have larger ratios of transition versus transversion mutations; these ratios far exceeded averages from the literature (28), indicated by a dashed blue line, and the transition-to-transversion ratio of 12B01 compared with a closely related strain, *V. splendidus* 12F01.

We found that two lineages (subsequently referred to as hypermutators) had accumulated a surprising number of mutations—more than 1,500 single-nucleotide mutations over an estimated 400 generations (e.g., lineages 2 and 6 in Fig. 1a; see Table S1 in the supplemental material). These mutation rates (3 to 4 mutations per generation) are 3 orders of magnitude higher than reported averages for bacteria (~0.003 mutations per generation) (2). All other lineages (subsequently referred to as mutators) still had mutation loads 2 to 3 orders of magnitude higher than expected (e.g., lineage 8 in Fig. 1a; Table S1). Lineages did not accumulate mutations evenly across selection rounds but instead did so sporadically (Fig. 1b). Previous studies have shown that mutations often follow a Poisson distribution, which assumes a stable average mutation rate (31, 32), but we found that in all lineages (including the hypermutators), the variance in the number of new mutations across selection rounds was far greater than that expected under a Poisson model (Fig. 1c), suggesting that the mutation rate in this strain varied substantially more than rates in other commonly studied bacteria, such as *E. coli* (31, 33, 34).

10.1128/mBio.02045-16.2 Table S1  Composition of SNP mutations accumulated across all experimental lineages. Download Table S1, XLSX file, 0.1 MB. Copyright © 2017 Chu et al. 2017 Chu et al. This content is distributed under the terms of the Creative Commons Attribution 4.0 International license.

The mutations in all lineages displayed a dramatic bias toward transition mutations, which accounted for 96% of observed mutations, or a transition-to-transversion mutation ratio of 22.6 (χ^2^ = 3461.67, df = 7, *P* < 10^−308^) (Table S1). Moreover, when rounds of selection generated more mutations, we observed even greater bias (ordinary least squares: *F* statistic = 29.68, df = 35, *P* < 10^−5^, *R*^2^ = 0.459) (Fig. 1d). Using a binomial model of transition frequency and a maximum-likelihood estimator, we found that mutations among our two hypermutator lineages were best described by a transition-to-transversion mutation ratio of 24.6, mutator lineages by 16.5, and all lineages by 22.8—values substantially higher than typically reported values for wild-type strains (2.1 to 3) (see Fig. S2a in the supplemental material) (35). These values are also much higher than the transition-to-transversion ratio of SNPs when comparing 12B01 with the closely related strain 12F01—which is 2.1 (71/105 SNPs) (Fig. 1d; Fig. S2a)—or with the more distantly related 13B01—which is 2.8 (30,975/42,012 SNPs). On the basis of these results, we speculated that all strains had developed a defect in DNA mismatch repair, which is known to produce similar, characteristic increases in mutation load and transition frequency (36, 37). In addition, we posited that this defect could be transient, given the extreme variability in mutation load across selection rounds.

10.1128/mBio.02045-16.3 Fig. S2  A novel mobile element excises from the host chromosome. (a) Normalized likelihood of binomial models with different transition frequencies given the mutation data. The null model is based on literature reports of average transition frequencies across multiple bacterial species (28). Lack of sequencing coverage indicated a 27-kb deletion shared by both hypermutator lineages. Reads mapping to this DNA region resulted from misalignment in genes with high similarity to other regions of the genome. (e) In *V. splendidus* 12B01, the orphaned start sequence to *mutS*—but not the mobile element-provided start sequence—matched the sequence of closely related isolates with no mobile element. (f) attP and attB sites of the mobile element revealed close homology. (g) qPCR results of the ratio of attB abundance during stationary and exponential growth (attB abundance during stationary growth divided by attB abundance during exponential growth) show that mobile element excision appears greater during stationary-phase growth. (h) Altered starburst morphology of hypermutator lineages grown on agar plates. Download Fig. S2, PDF file, 0.6 MB. Copyright © 2017 Chu et al. 2017 Chu et al. This content is distributed under the terms of the Creative Commons Attribution 4.0 International license.

### Excision of a mobile element results in *mutS* scarring and a hypermutator phenotype.

**FIG 2:**
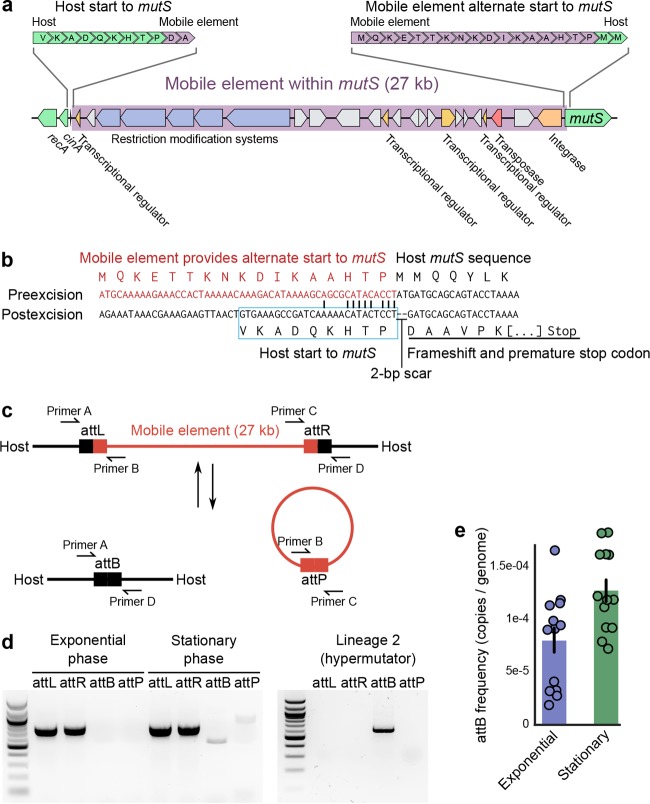
Excision of a mobile element within *mutS* disrupts the *mutS* genetic sequence. (a) We identified a mobile element adjacent to *mutS* that was missing in both hypermutator lineages. Further inspection revealed that, when present, the mobile element appeared integrated within the *mutS* sequence, separating the original host-encoded *mutS* starting sequence. (b) When integrated, the mobile element provided a new start and upstream regulatory region to the *mutS* coding sequence. After excision, the mobile element left a 2-bp frameshift deletion in the host’s *mutS* sequence, resulting in a premature stop codon. (c) We designed a PCR assay to detect the excision of this mobile element. When the mobile element is integrated into the host *mutS* sequence, the left (attL) and right (attR) attachment site junctions of the mobile element and host genome are amplified. When the mobile element is excised, the rejoined host *mutS* gene (attB) and the circular excised mobile element (attP) are amplified. Expected amplicon lengths: attL = 819 bp, attR = 836 bp, attB = 613 bp, and attP = 1,042 bp. (d) We found that in rich medium (LB), the mobile element excised itself at low frequency. Sanger sequencing of PCR products attB and attP confirmed the 2-bp frameshift deletion in the host *mutS* sequence and the transfer of these base pairs to a circularized mobile element. In hypermutator lineages (e.g., lineage 2), we could no longer detect the mobile element, only the scarred host *mutS* sequence. (e) qPCR assays indicated that the frequency of excision was approximately 1/10,000 genomes, with moderately higher excision frequency during the stationary phase.

From our whole-genome sequencing reads, we found that both hypermutator lineages shared a deletion of a 27-kb region adjacent to *mutS* (Fig. S2d). On closer inspection of the wild-type sequence, this deletion appeared to be a mobile element that resided within a conserved amino acid motif at the beginning of the *mutS* gene (Fig. 2a). The mobile element effectively provided a new translation start sequence and promoter region for *mutS*, which appeared to result in a functional *mutS* coding sequence. Upstream of the mobile element, we identified a putative N-terminal coding region for *mutS*. Unlike the starting *mutS* sequence provided by the mobile element, this orphaned *mutS* starting sequence matched that of closely related *Vibrio* strains, which suggests it is the ancestral, host-derived starting sequence of *mutS* (Fig. S2e). In hypermutator lineages—which had lost the mobile element altogether—we found that this host-derived start sequence was rejoined with the rest of the *mutS* sequence, but excision of the mobile element had also removed two additional base pairs, leaving a frameshift mutation, which resulted in an early stop codon and a disrupted *mutS* sequence (Fig. 2b).

We observed sequence similarity between the terminal ends of the mobile element and its insertion site in 12B01’s *mutS* sequence, which resembled the direct repeats of mobile elements that excise themselves by site-specific recombination (Fig. 2b; Fig. S2f). We therefore hypothesized that 12B01’s mobile element may also excise itself by this means into a circular DNA element. To test whether (i) the mobile element excised itself into a circular DNA product and (ii) whether it did so under conditions other than salt selection, we designed a PCR assay that amplified different sequences, depending on whether the mobile element was integrated within *mutS* or was excised as a circular DNA element (Fig. 2c).

We found that *V. splendidus* 12B01 exhibited low levels of mobile element excision during growth in standard rich medium (Fig. 2d; Fig. S2g). Lineages that had lost the mobile element (e.g., lineage 2) produced PCR products exclusively from the scarred *mutS* host sequence (Fig. 2d). DNA sequencing of each PCR product indicated that mobile element excision in the ancestral strain under standard laboratory conditions resulted in the same 2-bp frameshift mutation we observed in the hypermutators and that these 2 bp were located in a circular DNA element containing the mobile element.

On the basis of quantitative PCR (qPCR) results (see Materials and Methods), we estimated that the frequencies of excision in the original ancestral strain were 7.95 × 10^−5^ ± 1.25 × 10^−5^ (mean ± standard error [SE]; approximately 1/12,500 genomes) during the exponential growth phase and 1.27 × 10^−4^ ± 1.07 × 10^−5^ (mean ± SE; approximately 1/8,000 genomes) during the stationary phase (Fig. 2e; Fig. S2g). Thus, this mobile element appeared to have excised itself in a small fraction of the population during all stages of growth and perhaps did so slightly more often during the stationary phase (Student’s dependent *t* test, *t* = −8.36, *P* = 2.39 × 10^−6^).

### Mobile elements within *mutS* exist across *Vibrio* and *Betaproteobacteria* and *Gammaproteobacteria*.

To establish whether this mobile element was unique to *V. splendidus* 12B01, we looked for similar elements in other bacterial strains. Using the mobile element’s putative integrase as a search query, we performed a protein BLAST search of bacterial genomes to identify related integrases. We then screened for those related sequences also adjacent to *mutS*. From all bacterial genera that contained a genome identified by our search, we manually checked representative genomes to confirm that the integrase-like sequences were part of a putative mobile element present within the bacterial host *mutS* sequence. To determine that these elements had inserted themselves into host *mutS* sequences, we checked for upstream host-derived *mutS* start sequences homologous to *mutS* genes from other closely related strains (see Table S2 in the supplemental material).

10.1128/mBio.02045-16.4 Table S2  Mobile elements within *mutS* across *Betaproteobacteria* and *Gammaproteobacteria*. Shown are strains of *Betaproteobacteria* and *Gammaproteobacteria* that have related integrases—identified by BLASTP—adjacent to the *mutS* gene. Strains that were manually confirmed to contain a putative mobile element within *mutS* are noted. Download Table S2, XLSX file, 0.1 MB. Copyright © 2017 Chu et al. 2017 Chu et al. This content is distributed under the terms of the Creative Commons Attribution 4.0 International license.

**FIG 3:**
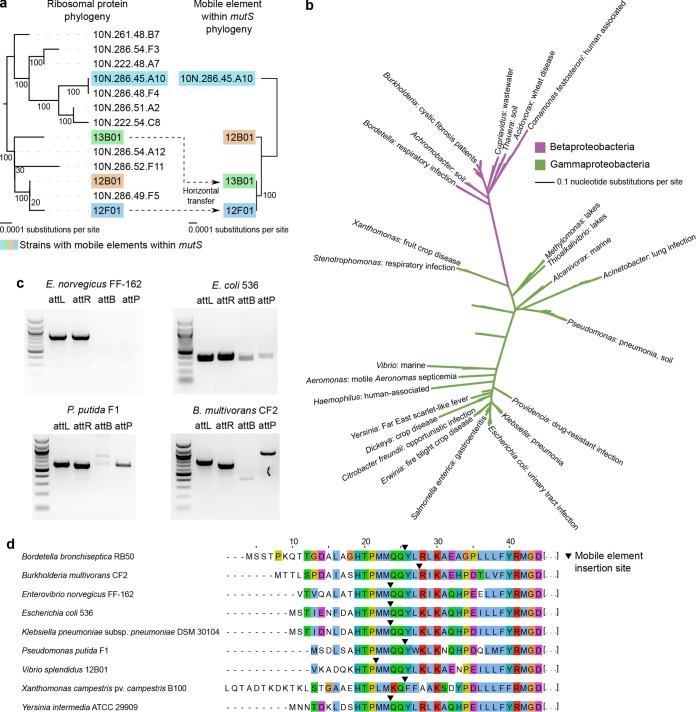
Mobile elements within *mutS* occur across *Vibrio*, *Betaproteobacteria*, and *Gammaproteobacteria*. (a) Phylogeny of close relatives of 12B01 (>98% similarity in 16S rRNA). Strains with mobile elements within *mutS* are not a monophyletic clade, and comparison between host phylogenies and mobile element phylogenies indicates that these elements have been horizontally transferred (dotted lines). (b) Broader BLAST searches identified other bacteria with mobile elements within *mutS*, including many human pathogens (Table S2). Phylogenetic tree built using 16S rRNA sequences. (c) Using a PCR assay similar to what we used for 12B01, we found mobile element excision in some, but not all, of a small subset of these bacteria when grown to the stationary phase. Sanger sequencing of the attB and attP PCR products from *E. coli* 536, *P. putida* F1, and *B. multivorans* CF2 indicated that mobile element excision in these bacteria did not result in any deletions or frameshift mutations. Expected amplicon lengths: *E. norvegicus* FF-162 attL = 894 bp, attR = 891 bp, attB (hypothetical) = 380 bp, and attP (hypothetical) = 1,405 bp; *E. coli* 536 attL = 404 bp, attR = 416 bp, attB = 393 bp, and attP = 427 bp; *P. putida* F1 attL = 601 bp, attR = 583 bp, attB = 596 bp, and attP = 588 bp; and *B. multivorans* CF2 attL = 811 bp, attR = 723 bp, attB = 451 bp, and attP = 1,083 bp. (d) These mobile elements were all integrated into the HTPMMQQ amino acid motif in MutS, although the precise location varied.

We initially confined our search to the genus *Vibrio*, identifying a number of strains with putative mobile elements in *mutS*; many of these were closely related to 12B01 (Fig. 3a; see Fig. S3a in the supplemental material). Among close relatives (>98% similarity in 16S rRNA), the phylogeny of hosts did not match the phylogeny of the elements. We found evidence for horizontal mobile element transfer between strains 12F01 and 13B01, which had nearly identical mobile element sequences (>99.99% nucleotide similarity for >19 kb [Fig. 3a; see Table S3 in the supplemental material]). It appeared to be a transfer of only the mobile element, as adjacent genes followed the overall divergence of the host strains (Fig. S3c and Table S3). Other, less closely related *Vibrio* strains had elements of various lengths and structures in *mutS*. For example, *Enterovibrio norvegicus* FF-162 appeared to have a 7-kb element with few genes (see Fig. S4a in the supplemental material). Using a PCR strategy similar to the one we used with 12B01, we detected no excisions in liquid FF-162 cultures (Fig. 3c), which could indicate (i) that the 7-kb element may have degraded over time, so that it could no longer excise itself from the host genome, or (ii) that its excision requires specific conditions we did not test.

10.1128/mBio.02045-16.5 Fig. S3  Phylogeny of *Vibrio* hosts and mobile elements. (a) Phylogeny of genomes from across the genus *Vibrio*, indicating in red those containing mobile elements within *mutS*. These elements were not contained within a monophyletic group. (b) Phylogeny of integrases from mobile elements within *mutS*. Elements are labeled by bacterial host genome and phylum and give evidence that these elements have been transferred horizontally. (c) In close relatives of 12B01, the phylogeny of proteins adjacent to the mobile element within *mutS* follow host phylogeny but not mobile-element phylogeny. For each gene, maximum likelihood trees show bootstrap support from 1,000 bootstraps as a percentage (54). The disagreement between trees built using host and mobile element genes indicates that only the mobile element was horizontally transferred between 12F01 and 13B01. Download Fig. S3, PDF file, 0.2 MB. Copyright © 2017 Chu et al. 2017 Chu et al. This content is distributed under the terms of the Creative Commons Attribution 4.0 International license.

10.1128/mBio.02045-16.6 Table S3  Percentage of identity matrices for genes from close relatives of 12B01. This table includes the 16S, *mutS*, *cinA*, *recA*, and mobile element integrase genes from four closely related strains of *V. splendidus* with mobile elements within *mutS*. Download Table S3, XLSX file, 0.1 MB. Copyright © 2017 Chu et al. 2017 Chu et al. This content is distributed under the terms of the Creative Commons Attribution 4.0 International license.

10.1128/mBio.02045-16.7 Fig. S4  Diagrams of mobile elements within *mutS*. (a) Elements in *E. norvegicus* FF-162, *E. coli* 536, *B. multivorans* CF2, and *P. putida* F1. (b) The HTPMMQQ motif is part of the mismatch-binding region of MutS. The region appears to interact with the DNA backbone in crystal structures. Pink arrows indicate the amino acid motif in a 3D crystal structure of *E. coli* K-12 MutS complexed with a G-T mismatch (38). Download Fig. S4, PDF file, 0.5 MB. Copyright © 2017 Chu et al. 2017 Chu et al. This content is distributed under the terms of the Creative Commons Attribution 4.0 International license.

We then broadened our search to all bacteria whose genome sequences are available. The most similar integrase sequences from our BLAST search (before screening for sequences near *mutS*) came from a diverse set of strains in *Betaproteobacteria* and *Gammaproteobacteria*. These sequences fell predominantly adjacent to *mutS*. For example, all of the 100 most-similar sequences from the full NCBI nucleotide database appeared in putative mobile elements within host *mutS* sequences. The bacteria containing these sequences included opportunistic pathogens of human lungs, skin, and urinary tract, as well as pathogens of major crops (Fig. 3b; Table S2).

The putative mobile elements varied in length (4 kb to >150 kb) and gene content. For example, some of the elements appeared to be prophages and contained multiple open reading frames annotated to phage proteins (e.g., *Burkholderia multivorans* CF2 [Fig. S4a]). A phylogeny of putative mobile elements based on the elements’ integrase genes indicated that elements within bacteria from the same phyla largely clustered together (Fig. S3b). But as among close relatives of 12B01, the phylogeny of the elements often did not reflect the phylogeny of the host bacteria, implying possible horizontal transfer of mobile elements between diverse bacteria (Fig. S3b). The unifying feature of these elements was the location of their insertion sites near a well-conserved amino acid motif (HTPMMQQ) that helps MutS bind mismatched DNA base pairs (38) (Fig. 3d; Fig. S4b).

We experimentally tested a subset of strains outside *Vibrio* (*E. coli* 536, *B. multivorans* CF2, and *Pseudomonas putida* F1) for element excision and frameshift mutations. We chose these strains because the hosts are scientifically important and phylogenetically diverse and the elements are structurally disparate (Fig. S4a). Using a PCR assay design similar to what we used for *V. splendidus* 12B01, we detected excision in all three strains (Fig. 3c). An additional PCR product for the postexcision sequence (attB) of *P. putida* F1 came from a nonspecific binding site, as confirmed by DNA sequencing. Sequencing of the postexcision sequences indicated that, unlike in 12B01, excision did not leave a 2-bp deletion in *mutS*. We therefore inferred that, although the translation start sequence and promoter region had changed, *mutS* maintained an in-frame coding sequence whether these mobile elements were integrated or excised.

## DISCUSSION

### A mobile element alters the mutation rate in *V. splendidus* 12B01.

Although theoretical and empirical evidence suggests that many bacteria have variable mutation rates, we know of few mechanisms for generating this variability. Using whole-genome sequencing, we discovered a novel mechanism of hypermutation in *V. splendidus* 12B01—a mechanism brokered by the excision of a mobile element found within a conserved amino acid motif near the start of *mutS*. By residing in the start of this gene, the mobile element might regulate *mutS* in two ways. One way is to provide, when integrated, an entirely new amino acid start sequence and upstream regulatory region for *mutS*. This mobile element-provided regulatory region might respond to different environmental and cellular cues than the original host sequence does, potentially altering *mutS* expression patterns. Another way, exhibited in our hypermutator lineages, is to inactivate the *mutS* coding sequence by excision and a 2-bp deletion. In 12B01, the introduction of a frameshift mutation by mobile element excision adds a new mechanism for *mutS* to act as a contingency gene (27). In this case, mutability in *mutS* is brokered by the activity of a mobile element, not constitutive or loss-of-function mutations, thereby allowing 12B01 to rapidly switch to a hypermutator genotype. Such cooption of prophages or mobile elements for regulating host genes has been called “active lysogeny” and might be widespread across bacteria (39).

One plausible model consistent with our mutation results is one in which the mobile element within *mutS* reversibly excises itself from the genome, producing a variable and characteristic mutation rate. In our mutator lineages that retained the mobile element, mutation rates were higher and more variable than would be expected if their mutation rate were stable. This variance stemmed primarily from transition mutations, which are more affected by changes in *mutS* than are transversion mutations (Fig. 1c) (20). Further, although our mutator lineages retained the mobile element, they showed ratios of transition to transversion mutations very similar to those of our hypermutators, particularly in rounds of selection with more mutations. These ratios were also much higher than those between 12B01 and other closely related isolates of *V. splendidus* (Fig. 1; Fig. S2a). Thus, it is possible that 12B01’s mobile element may have temporarily excised itself during the growth of our mutator lineages, giving rise to characteristic mutation profiles, but then reintegrated itself shortly afterward, limiting the total number of mutations.

Our results do not directly demonstrate reintegration of this mobile element after excision. Because we were unable to genetically modify the bacterium or isolate the mobile element in its circular form, we could not introduce genetic reporters or transform naive strains. We can, however, see evidence from isolates closely related to 12B01 that this element is capable of transferring horizontally between carrier and naive cells and inserting itself into a host genome—also consistent with the hypothesis that these mobile elements can reintegrate into the host genome (Fig. 3a).

One caveat is that like our mutator lineages, our hypermutators had variable mutation rates. Because our hypermutator lineages appeared to have lost the mobile element within *mutS* (Fig. 2), the variability in mutation rates in these lineages cannot be explained by excision and reintegration of this element. It could be generated by a downstream effect of losing mismatch repair, which might destabilize the cell, either directly or because of the high mutation loads it produces. Indeed, we noticed that hypermutators grew slowly and had a unique starburst growth pattern (Fig. S2h). Other studies performing whole-genome sequencing on *E. coli* mutators with fixed defects in mismatch repair also found evidence that mutators had larger than expected variance in mutation rate (40) (see Fig. S5 in the supplemental material), suggesting that variable mutation loads may be common among bacterial mutators.

10.1128/mBio.02045-16.8 Fig. S5  *Escherichia coli* mutators also exhibit higher variance than nonmutator strains. Like Fig. 1c, colored bars represent the variance in the number of mutations gained during each time interval versus the Poisson model expectation. Because the sampling did not follow a regular generational interval, we split data before analysis into genomes that were sequenced 500, 5,000, and 10,000 generations apart. Nonmutators largely followed a Poisson model, but mutators exhibited much higher variance in mutation rate compared with the Poisson model expectation. This is an original figure and analysis using data from reference 40. Download Fig. S5, PDF file, 0.1 MB. Copyright © 2017 Chu et al. 2017 Chu et al. This content is distributed under the terms of the Creative Commons Attribution 4.0 International license.

Alternatively, our observed mutation profiles in all lineages might be driven by an unidentified mechanism, such as the activity of error-prone polymerases or downregulation of *mutS* by the bacterial SOS response. This unidentified mechanism might even have been caused by some of the mutations gained during the experiment, although few mutations were shared across all lineages (see Table S4 in the supplemental material). It remains unclear what other mechanism would result in the variable and transition-dominated mutation patterns we observed.

10.1128/mBio.02045-16.9 Table S4  Genes with SNPs in multiple lineages after five rounds of salt selection. Download Table S4, XLSX file, 0.1 MB. Copyright © 2017 Chu et al. 2017 Chu et al. This content is distributed under the terms of the Creative Commons Attribution 4.0 International license.

In addition, we found weak evidence that the mobile element excises itself from *mutS* more often during the stationary phase, hinting that the element’s activity might fit into a stress-induced mutagenesis model. But the similar excision rates under presumably less stressful conditions (i.e., log-phase growth) suggest that this element may also excise itself stochastically (Fig. 1). We believe that the role of 12B01’s mobile element may better align with previously described models where environmental change selects for or against low-frequency mutators in a population—in other words, stress-selected mutagenesis (12).

In the case of 12B01, mutators arise not from a constitutive or loss-of-function mutation (as in previous studies [11–13]), but from the stochastic, potentially reversible excision of a mobile element. Subpopulations of mutators might be selected for under certain conditions, such as the hypersaline environment of our experiments, and provide a source of genetic diversity. Our results do not preclude the possibility that excision of this mobile element is regulated by an unknown cellular cue, perhaps linked to stress. For example, studies in other bacteria have identified mechanisms that link mutation rate (41) and mobile element recombination (42) to the SOS response.

From the mobile element’s perspective, the phenomena we observed might have several benefits. The most compelling benefit might be that the bacterial host depends on the mobile element to prevent rapid accumulation of deleterious mutations. Any bacterium that permanently loses the mobile element, like our hypermutators, would bear a heavy cost in fitness. Although our experimental selection favored two hypermutator lineages, we expect that the burden of deleterious mutations would eventually decrease fitness, particularly in natural environments.

### Widespread mobile elements within *mutS* in *Betaproteobacteria* and *Gammaproteobacteria*.

Although *mutS* is well conserved (43) and well studied, the presence of putative mobile elements inserted into—rather than adjacent to—this gene has not been reported (30). We identified putative mobile elements within *mutS* in hundreds of *Betaproteobacteria* and *Gammaproteobacteria* from human and environmental sources, including important human and agricultural pathogens (e.g., bacteria that cause pneumonia, urinary tract infections, and fire blight crop disease). During our search, we found that the most similar integrase sequences came from mobile elements within *mutS*. This similarity in integrase sequence and putative insertion site may suggest that these elements share common ancestry, although the complex and mosaic structure of mobile elements makes detailed phylogenetic inference difficult. Our phylogeny built using only the integrase genes in these elements hinted that these elements are horizontally transferred between diverse bacteria, suggesting that perhaps one or multiple families of putative mobile elements have taken advantage of a well-conserved amino acid motif in the *mutS* gene.

Although we observed mobile element excision in *E. coli* 536, *B. multivorans* CF2, and *P. putida* F1, we did not find that excision left a scar in these bacteria, indicating that scarring might be specific to *V. splendidus* 12B01. Element excision in these bacteria might nevertheless alter *mutS* expression in these bacteria. As in 12B01, the integrated mobile element in these strains provides an independent start sequence and promoter region for each bacterium’s *mutS* gene, but instead of inactivating it, excision returns *mutS* to control of the bacterium’s own *mutS* promoter. Thus, excision switches control of *mutS* between two regulatory regions, which might encode different levels of expression or respond to different environmental and cellular cues. Further study of these strains could reveal whether these elements alter mutation rates and drive adaptation in bacteria other than *V. splendidus* 12B01.

## MATERIALS AND METHODS

### Serial salt selection.

As part of a larger effort to characterize the microbial ecology of the coastal ocean, we isolated *V. splendidus* 12B01 from seawater collected at the Plum Island Estuary Long-Term Ecological Research site (44) (see Table S5 in the supplemental material). Cultures were grown at room temperature with shaking (200 rpm) in lysogeny broth (LB) supplemented with 0.5 M NaCl unless otherwise specified.

10.1128/mBio.02045-16.10 Table S5  Strains and primers used in this study. Download Table S5, XLSX file, 0.1 MB. Copyright © 2017 Chu et al. 2017 Chu et al. This content is distributed under the terms of the Creative Commons Attribution 4.0 International license.

We prepared solid medium plates with a gradient of salinity so that we could select for mutants of 12B01 with higher than normal salt tolerance. We made these plates by first elevating one edge of a 241 mm by 241 mm by 20 mm square culture plate by 5 mm and pouring a wedge-shaped, high-salinity (2.2 M NaCl) layer of LB. Once this high-salinity layer solidified, we then placed the plate on a flat surface and poured a wedge-shaped layer of LB without additional NaCl. We allowed the plates to equilibrate for 48 h to allow diffusion from the high-salinity layer to the low-salinity layer, thereby establishing a salinity gradient.

To grow and select salt-tolerant mutants, we spread overnight cultures of *V. splendidus* 12B01 on one-half of a salinity gradient plate and incubated the plates for 48 h. We picked colonies that grew at higher salinities than the majority of cells and restreaked them at the same gradient position in the other half of the culture plate to confirm salt tolerance and prevent contamination with cells that were not salt tolerant. To further eliminate contamination and maintain selection pressure, we restreaked these colonies again on LB agar plates supplemented with salt of increasing concentrations for each selection round (round 1, 0.7 M total NaCl; round 2, 0.9 M; round 3, 1.0 M; round 4, 1.1 M; round 5, 1.2 M). We then picked colonies from these final plates and used them to inoculate liquid LB of the same salinity as the plate medium from which we picked the colonies. After incubating these liquid cultures overnight, we used them to extract DNA for genomic analysis, stored a subsample at −80°C with 15% glycerol, and started the next round of selection on new salinity gradient plates. In total, we collected and analyzed eight independent lineages of 12B01 over five rounds of salt selection. To estimate the number of generations per round of selection, we counted the number of cells within an average colony grown on solid medium or in liquid medium (~80 generations per round of selection).

### Whole-genome sequencing and analysis.

To analyze genome-wide mutations among these isolates, we used a Qiagen Genomic-tip 500/G kit to extract DNA from overnight liquid cultures. We fragmented genomic DNA by sonication with a Bioruptor (Diagenode) with 30-s cycles for 18 min. Illumina whole-genome sequencing libraries were prepared at MIT’s BioMicroCenter and sequenced on a single Illumina HiSeq lane using 40-bp paired-end reads. Average coverage across all lineages was 35× ± 16× (mean ± standard deviation [SD]).

We searched for SNP mutations as indicators of salt adaptation, using a custom Galaxy pipeline (45). We cleaned sequencing reads using FASTQ Groomer (46) and aligned reads to the wild-type reference genome of *V. splendidus* 12B01 (NCBI genome assembly GCA_000152765.1 [Gordon and Betty Moore Foundation Marine Microbiology Initiative]) using Bowtie 1.0.0 (47). We identified fixed SNPs (more than 80% of reads) using SNP Finder (46) and filtered these SNPs for ambiguous or erroneous mapping, in particular, reads mapping to the ends of contigs and deleted regions. By checking for shared SNPs, we confirmed that each strain from each selection round was the progeny of the previous selection round. To check that sequencing coverage across our isolate library did not bias our SNP calling procedure, we confirmed that read coverage did not trend with the number of SNPs called (Fig. S2). We classified each SNP as a transition or transversion, coding or noncoding, and synonymous or nonsynonymous mutation. To identify deletion mutations, we searched alignments for regions of the genome that lacked read coverage for more than 100 bp.

To model the transition frequency best describing our SNP mutation data, we used a binomial model to calculate the likelihood (*P*) of each data point (e.g., *x* transition mutations out of *y* total mutations during a given selection round for a given lineage of 12B01) given a probability (*p*_*ti*_) that a mutation will be a transition:

$$P\left[\text{binomial​}\left(k\;\text{transitions},n\text{ total SNP mutations},p_{ti}\right)\right]=\left({}_{k}^{n}\right)\text{​}p_{ti}{}^{k}\text{​}{\left(1-p_{ti}\right)}^{n-k}$$

Thus, the probability of the model given *p*_*ti*_ is the product of the probability of each data point:

$$P\left(\mathrm{model}\;p_{ti}\text{​}|\text{data}\right)=\prod_{i}^{j}\text{​}\;\left({}_{k}^{n}\right)p_{ti}{}^{k}\text{​}{\left(1-p_{ti}\right)}^{n-k}$$

### PCR and qPCR assays.

To establish whether mobile elements within *mutS* excised from the host chromosome, we used Kapa 2G Fast Ready mix for all PCRs, applying the manufacturer’s suggested reaction settings. We used Kapa SYBR Fast qPCR master mix for all qPCR experiments on a Roche LightCycler. To measure the frequency of mobile element excision, we employed a previously reported qPCR strategy (48). We created a dilution series of standards using DNA from lineage 2, which had 100% excision. We designed qPCR primers for attachment site attB (attB_12B01_qpcr145_fwd and attB_12B01_qpcr145_rev) and a nearby (~10-kb distant) genomic control locus (ctrlL_12B01_qpcr145_fwd and ctrlL_12B01_qpcr145_rev) (see Table S5 in the supplemental material). We then compared abundance measurements of attB and ctrlL from each of our samples against our 100% excision standards to obtain a relative frequency of mobile element excision.

### Phylogenetic analysis.

To search for other bacteria with similar mobile elements, we used the amino acid sequence of the integrase from 12B01’s mobile element as a query for TBLASTN and BLASTP searches of the National Center for Biotechnology Information’s (NCBI) nucleotide and protein databases for similar integrases. For each hit with an E value of ≤1 × 10^−6^, we checked whether the hit lay within 300 bp of *mutS*. For a phylogenetically diverse subset of these strains, we manually checked whether the integrase was part of a putative mobile element that orphaned an original *mutS* starting sequence (Table S2). To test for mobile element excision in a subset of these strains, we designed PCR assays as for 12B01 (Table S5).

To visualize the phylogenetic distribution of these elements, we first built a tree for 530 *Vibrio* genomes based on 52 ribosomal proteins that are well conserved across this genus (49). To improve the resolution of this tree for the clade of 12B01’s close relatives, we trimmed the multiple-sequence alignment using trimAl (50). We used iTOL to plot the tree and annotate the presence of mobile elements within *mutS* (51). We aligned integrase genes from the *mutS* mobile elements of four *Vibrio* strains closely related to 12B01 and inferred a neighbor-joined phylogeny of these genes using Clustal Omega (52). For strains across *Betaproteobacteria* and *Gammaproteobacteria* with mobile elements within *mutS*, we downloaded each genome from NCBI and extracted each strain’s 16S rRNA sequences using RNAmmer (53). We aligned 16S rRNA sequences from each strain using Clustal Omega (52) and inferred a maximum likelihood tree using RAxML with 1,000 bootstraps (54).
