# Supplementary material for: A Mobile Element in mutS Drives Hypermutation in a Marine Vibrio
Source: mBio. 2017 Feb 7;8(1):e02045-16. doi: 10.1128/mBio.02045-16 (PMC5296598; doi:10.1128/mBio.02045-16)
Supplement: Fig. S1 [file mbo001163141sf1.pdf]

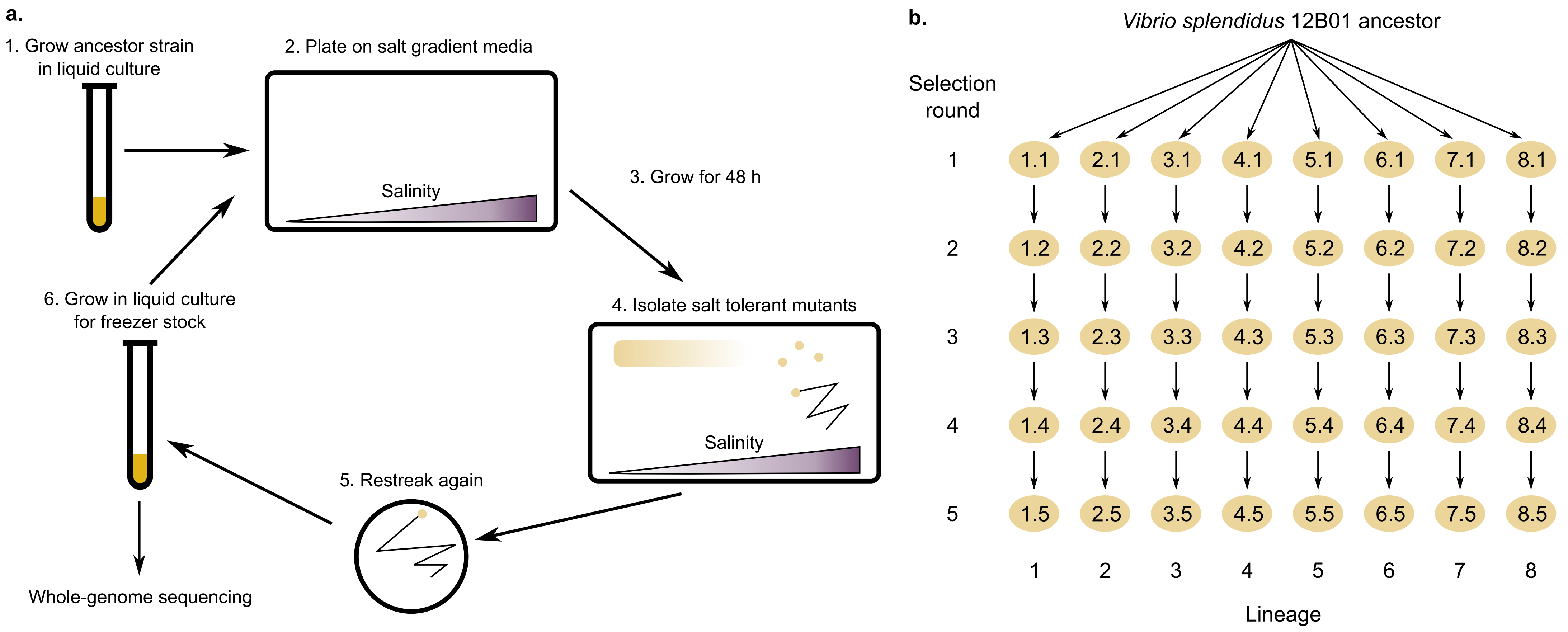
a.
1. Grow ancestor strain in liquid culture
2. Plate on salt gradient media
Salinity
3. Grow for 48 h
4. Isolate salt tolerant mutants
Salinity
5. Restreak again
6. Grow in liquid culture for freezer stock
Whole-genome sequencing
b.
Vibrio splendidus 12B01 ancestor
Selection round
1
2
3
4
5
1.1 2.1 3.1 4.1 5.1 6.1 7.1 8.1
1.2 2.2 3.2 4.2 5.2 6.2 7.2 8.2
1.3 2.3 3.3 4.3 5.3 6.3 7.3 8.3
1.4 2.4 3.4 4.4 5.4 6.4 7.4 8.4
1.5 2.5 3.5 4.5 5.5 6.5 7.5 8.5
1 2 3 4 5 6 7 8
Lineage
