# Supplementary figures and images for: A Mobile Element in mutS Drives Hypermutation in a Marine Vibrio
Source: mBio. 2017 Feb 7;8(1):e02045-16. doi: 10.1128/mBio.02045-16 (PMC5296598; doi:10.1128/mBio.02045-16)

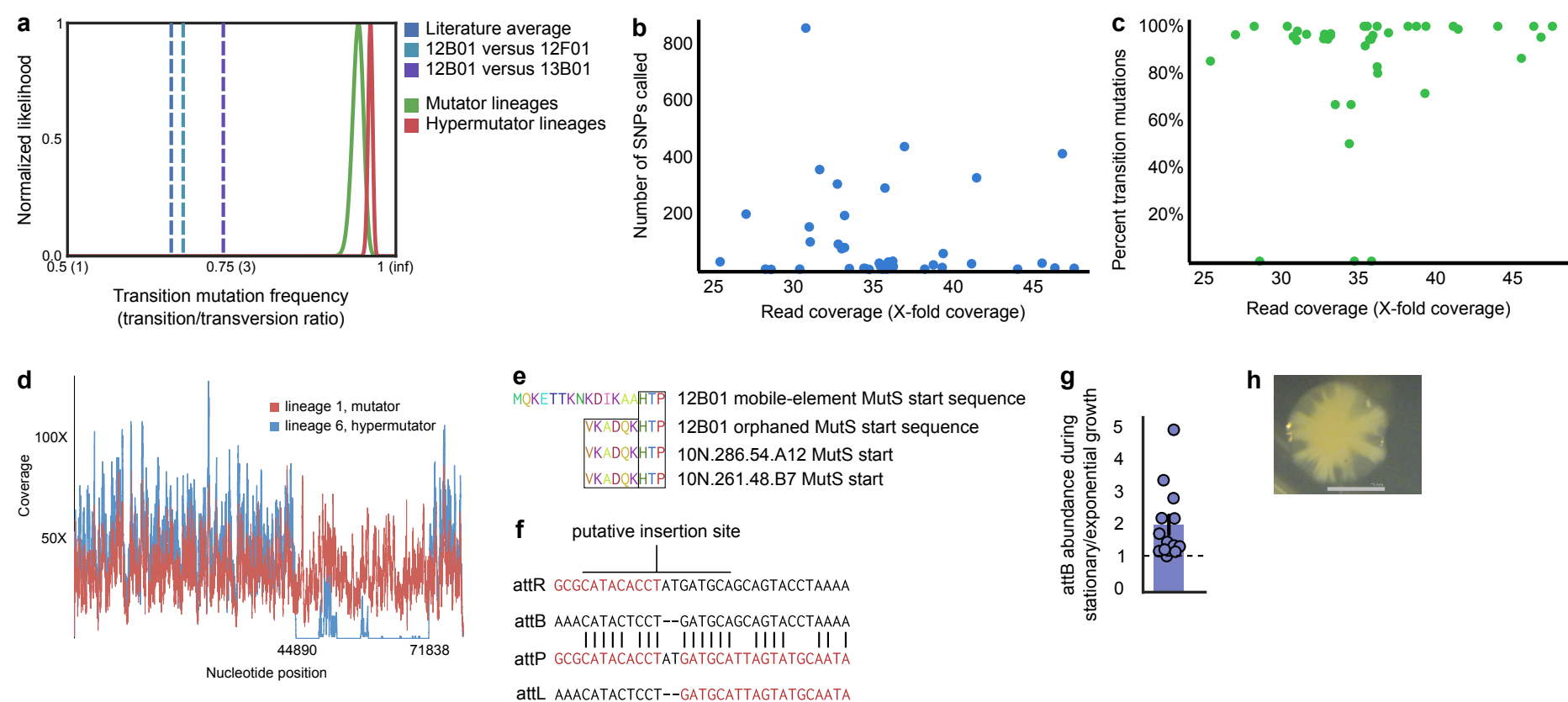

Supplement: Fig. S2 [file mbo001163141sf2.pdf]

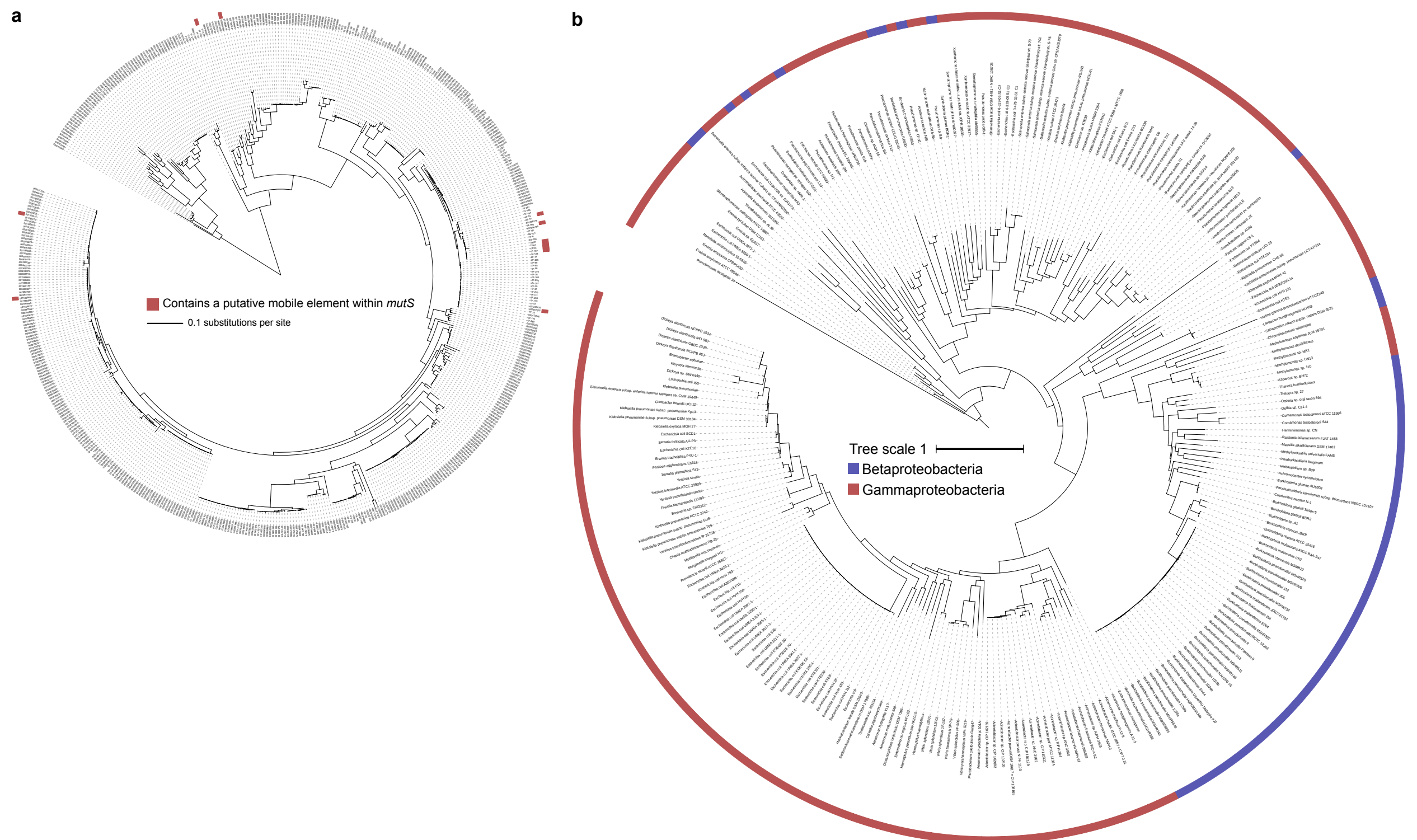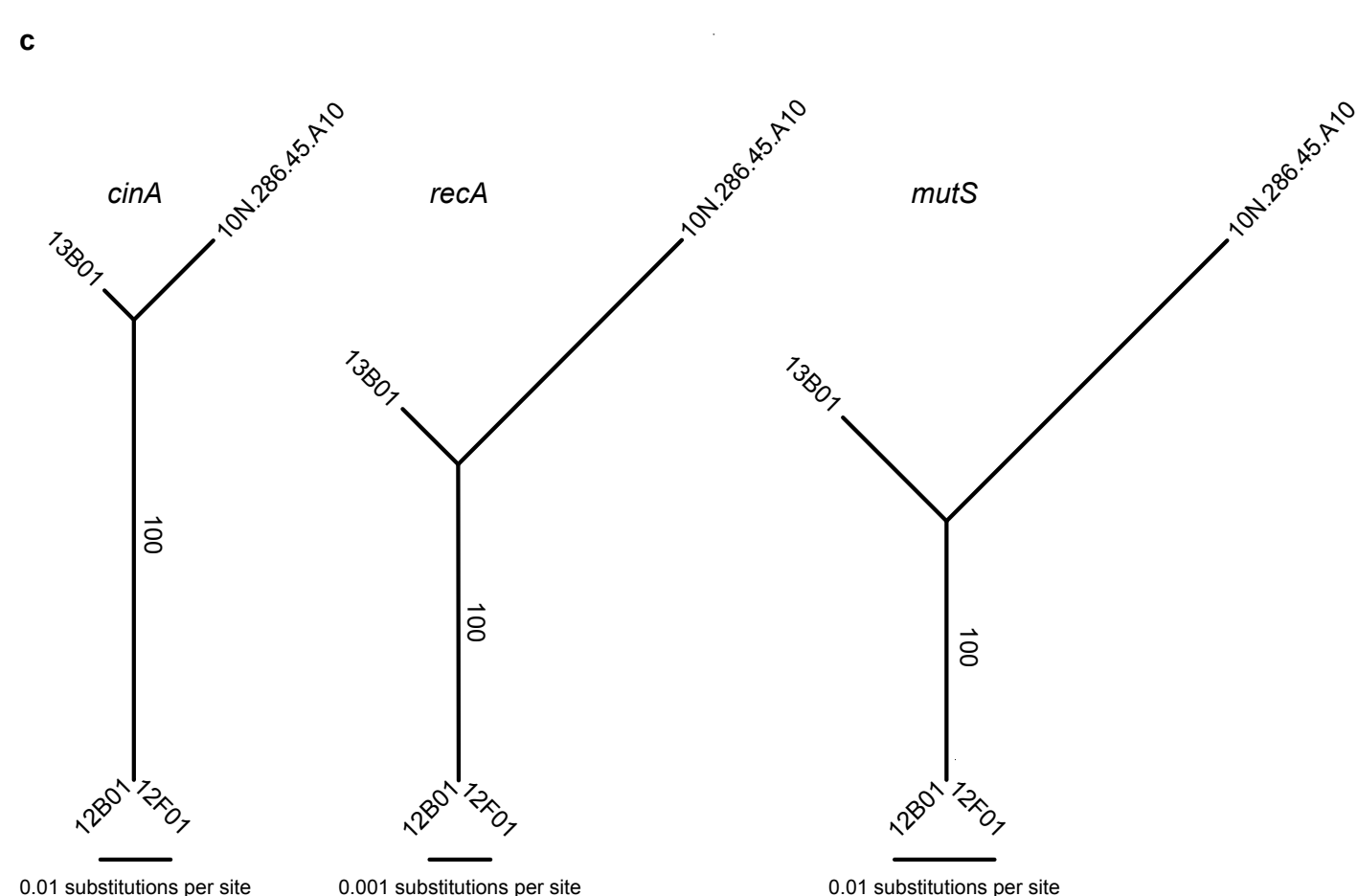

Supplement: Fig. S3 [file mbo001163141sf3.pdf]

**a**

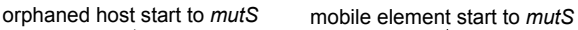

***Escherichia coli* 536**

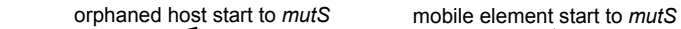

***Pseudomonas putida* F1**

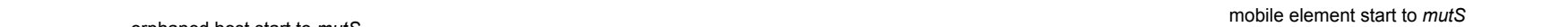

***Burkholderia multivorans* CF2**

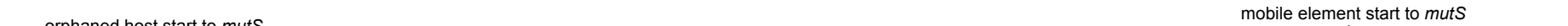

**b**

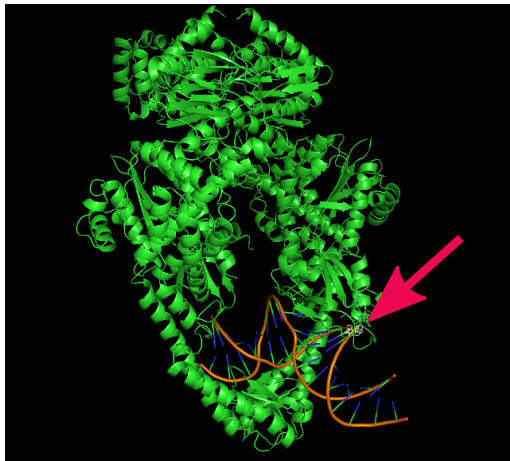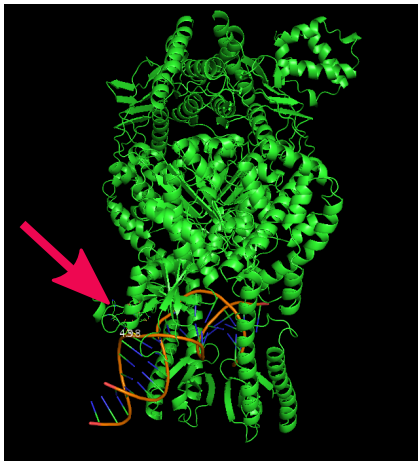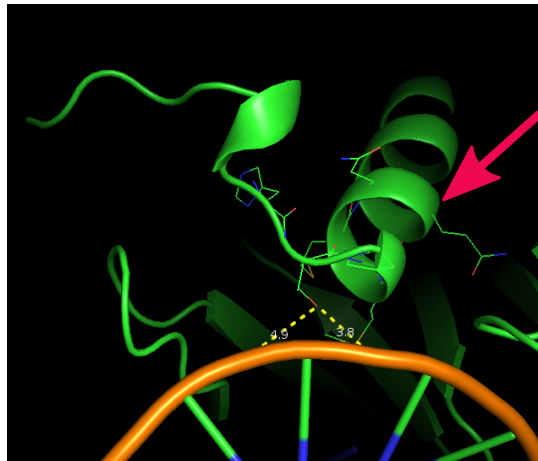

Supplement: Fig. S4 [file mbo001163141sf4.pdf]

**a**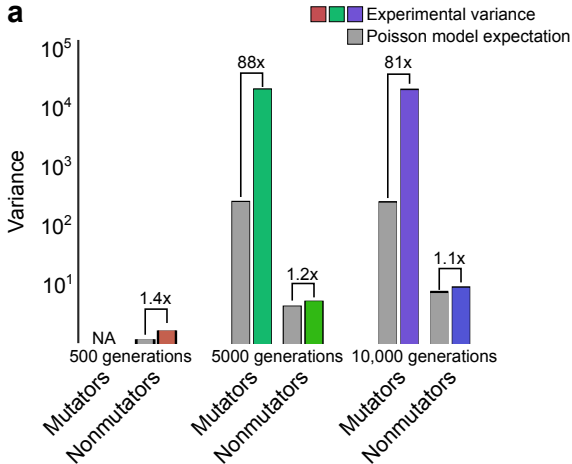

Supplement: Fig. S5 [file mbo001163141sf5.pdf]
